# Autistic people and life experiences: the role of student skills and support

**DOI:** 10.3389/fpsyt.2026.1733550

**Published:** 2026-04-10

**Authors:** Leslie A. Shaw, Katherine R. Brendli Brown, Hassan Enayati

**Affiliations:** 1 Yang Tan Institute on Employment and Disability, Cornell University, Ithaca, NY, United States; 2 Abt Global, Boston, MA, United States

**Keywords:** autism, independent living, life experiences, student skills, support, transition

## Abstract

**Introduction:**

Studies examining quality of life outcomes have shown that Autistic people experience lower scores across domains (e.g., physical, psychological) compared to other groups, with their lowest scores consistently reported in social functioning. However, to date, little is known about different adult life experiences that Autistic adults have beyond the typically tested outcome of living independently in the community, which may not be a desired outcome culturally for all Autistic people, particularly outcomes that could lead to improved social functioning, like being in relationships.

**Methods:**

Survey data were collected from a nationally representative sample of 272 U.S. residents with autism. Respondents were asked about secondary school transition activities, support, and later life experiences of moving into their own home, falling in love, getting married, pregnancy, becoming a caregiver, and being arrested.

**Results:**

Logistic regression results indicated goal setting and psychological empowerment were significant predictors of all adult life experience outcomes tested, and autonomy/decision-making predicted moving into one’s own home and falling in love. Social skills predicted both falling in love, getting married, and being arrested. For support, the number of people supporting the Autistic youth was related to increased odds of falling in love.

**Discussion:**

While transition programs are typically focused on furthering education and employment success in later life, skills taught to students to support those outcomes also relate to a broader range of life experiences for Autistic youth and adults. Programs to support Autistic students and youth should include, at a minimum, activities that build goal setting and psychological empowerment skills in hopes that Autistic adults can experience a fulfilling life of their choosing.

## 1 Introduction

A number of literature reviews on quality of life for Autistic people have been conducted over the years (1–4). A common finding has been lower quality of life scores across multiple domains (e.g., physical, psychological) for Autistic people when compared to other groups (e.g., typically developing peers), with the lowest scores related to social functioning. Yet, despite the robust evidence on this topic area, there remains a limited understanding of the extent to which Autistic adults have different types of social experiences (e.g., relationships, caregiving) after high school similar to neurotypical peers (5–7).

In the postsecondary transition literature, the most frequently researched life experience is independent living (8–10), which many studies describe as quality of life, among other conceptualizations. To explain, two systematic literature reviews on predictors described independent living as it was conceptualized in the included studies. A few studies in the first systematic literature review explored high quality of life as an outcome, which the systematic review conceptualized as independent living (11). A few studies in the second systematic literature review explored quality of life (which they deemed as socialization), participating in the community, or “living on own, with a roommate or spouse, in college housing or dormitory, or military housing” (p. 9), which this review also conceptualized as independent living (12). This reflects the diverse ways in which researchers have interpreted independent living in the literature.

Regardless of its conceptualization, research findings over the past few decades tell us that independent living outcomes (i.e., living away from one’s natal family) for Autistic people are generally low on an international scale. For Autistic youth, the final wave of the National Longitudinal Transition Survey-2 (NLTS2), collected in 2009, indicated that only 16.6% of Autistic youth were living independently (10). For Autistic adults who use adult developmental disability services and participated in the National Core Indicators program in 2012-2013, the independent living rate was 8% (13), and for a sample Autistic adults, many with a co-diagnosis of intellectual and developmental disability, living in the western United States (U.S.), 13% lived in their own home or apartment (14). In 2009, 9% of a sample of Autistic people from Australia reported living independently (15). An outlier to observations in other samples, 65.1% of a sample of Autistic adults in the United Kingdom (UK) reported not living with their family of origin, a spouse, or partner (16), a category that could have included living in a residential home or other supportive environment in addition to living independently. When conceptualized as the autonomous behavior of living on one’s own, independent living may not be a desired outcome for some people based on individual and family preferences or cultural values (17). Thus, it’s important to consider a range of activities a person may want or be interested in experiencing after high school.

Rates of different types of other life experiences have been collected both in the transition literature and quality of life studies, with social activities ranging from eating out and getting together with friends to getting married. A sample of Autistic, Australian adults were asked about a range of social outings, like attending a sporting event, going to church, or visiting a park, and the average number of social activities adults participated in over the previous three months was 4.25 out of 9; the most frequent outing was going to an eating or drinking establishment, reported by 81% of the sample (15). In the last wave of the NLTS2 sample, participants with disabilities spent on average 2.70 days a week with friends (8). Over half of Autistic adults in the western U.S. reported spending time with peers each week (53%) and were involved in organized groups (61%) (14). Relationship status varied across samples. In a sample of Autistic, Taiwanese adults, all Autistic adults aged 20 to 38 reported that they were single (18). In a UK sample, 40.8% reported that they were currently in a relationship (16). Lastly, in a U.S. sample of adults in a study that examined well-being, friendship, and loneliness, 22.2% were married, 2.8% were in a romantic relationship, and 6.5% were divorced; and 60.2% reported having a close or best friend (19). Thus, largely, rates of social outings and connections more broadly are low for Autistic young people and adults, similar to rates of independent living.

Across studies documenting rates of life experiences, little is known about the effects of personal characteristics or adaptive behavior related to those experiences, except for independent living. Autistic participants were compared to participants diagnosed with mental retardation (now referred to as ID), emotional disturbance, and learning disability and were found to be less likely to live independently compared to these groups. Identifying as African American decreased odds of living independently in comparison to participants identifying as White. Parental income, ability to converse well, and functional skills were found to be related to increased odds of living independently (10). Age was related to increased odds and more severe intellectual disability were related to decreased odds of living independently for National Core Indicators participants (13). One NLTS2 study did examine the effects of personal characteristics on social life experiences and found increased odds of participating in activities outside of classes or school for Autistic youth who identified as African American versus White. This was also true for those with a case manager and higher family income (20).

In 1983, the U.S. Office of Special Education Rehabilitative Services published a policy document that described a model for the successful transition of youth with disabilities from high school to employment. This work provided the foundation for the theory that transition programming that is personalized to the individual is related to postschool success in the domain of work and as a means to fully participate in the community (21). Educators need to provide effective transition programs for students with disabilities so they can be successful and participate in life like their nondisabled peers (22). More recently, three systematic reviews and one meta-analysis of secondary transition literature have led to the identification of 23 in-school transition experiences that predict postschool success in the multiple areas of education, employment, and/or independent living for students with disabilities (11, 12, 23, 24), which were defined through a separate Delphi study (25). These predictors range from typical career development activities and inclusion of students with disabilities in general education to student support and skills. However, few of these studies exclusively target or include Autistic youth in their samples, although the number of studies has grown in recent years (26).

Most findings regarding independent living also come from articles identified in a systematic literature review published in 2009 (11), which reported on studies conducted from 1984 to March of 2009 that met inclusion criteria (12). The one exception is research conducted on three domains of self-determination: psychological empowerment (i.e., belief in the relationship between actions and outcomes), autonomy (i.e., act according to own preferences, interests, and abilities without external influence), and self-realization (having an understanding of one’s strengths and support needs) (8). All three domains predicted independent living for all students with disabilities. In that same study (8), getting together with friends each week was used as a measure of social engagement for all youth with disabilities with no self-determination constructs significant predictors of this outcome, a sample that contained an unspecified number of Autistic participants.

Experiencing life like nondisabled peers can also include benign or negative interactions, like interactions with police. Data from the NLTS2 indicate that by age 22, 19.5% of Autistic youth had been questioned by police. Only 5% had been arrested and being a female was related to reduced odds of being questioned (27). In a study focused on Canadian Autistic youth in one province, by age 18, 20.3% of Autistic youth had some type of police involvement. In this case, police involvement was defined as contact but not categorized by type of involvement (e.g., taking statements from the youth as a victim, questioned as a perpetrator of a crime (28). In both samples, having a history of aggression or externalizing behavior was related to interactions with the police and arrest (27, 28).

The rates of independent living and other life experiences in studies with U.S. samples (8, 10, 13, 14) and an Australian sample (15) were based on participants diagnosed with versions of the Diagnostic Statistical Manual of Mental Disorders (DSM) prior to version 5 (DSM-5). The diagnostic criteria for autism were first introduced in version 3 of the DSM and expanded to include Aspergers diagnosis in version 4. The DSM-5 modified the diagnostic criteria to place autism, Aspergers, and pervasive developmental disorders under the single diagnosis of autism spectrum disorder (29). With a prevalence rate that was 1 in 68 in 2010 to now 1 in 31 in 2022 (30), the U.S. and Australian samples would likely be larger and more diverse if those studies were conducted this decade, and the independent living rates would also likely change given the changed diagnostic criteria. Given the lack of recent research on independent living and life experiences more broadly for Autistic young people, we examined predictors of six types of life experiences, including falling in love, getting married, pregnancy, being a caregiver, moving into one’s own home, and being arrested. Moving into one’s own home was selected because the broader construct of independent living is considered a primary outcome in transition research (11, 12) and is a goal area in line with transition planning requirements according to the Individuals with Disabilities Education Act (31). We explored three outcomes related to social and sexual relationships given that autism is defined in part as having difficulty with social interaction and communication (32). It is important to note that the selected life experiences in our research (e.g., falling in love, marriage, pregnancy, living away from the natal family) are not universal “markers” of a fulfilling life, but rather possible experiences whose meaning varies across preferences and contexts. All survey items were reviewed, with feedback obtained, by a subject matter expert panel consisting of people with lived experience and expertise in autism. We aimed to answer the following research questions:

1. To what extent do Autistic people have common life experiences?
2. How are demographics and proxies for adaptive behavior (physical health needs, mental health needs, and service receipt) related to life experiences?
3. What social supports and student skill experiences are related to life experiences?

## 2 Materials and methods

### 2.1 Procedure and participants

Throughout the development of the survey, the research team worked with a subject matter panel that was comprised of Autistic individuals and those with expertise in education and employment for Autistic people. The subject matter experts provided input on the survey design and consent forms as well as resources that were developed from findings. To assess the degree to which Autistic people across the U.S. have common life experiences and what skills were related to these experiences, researchers engaged Ipsos, a survey company, to identify KnowledgePanel participants who reported they had been diagnosed with autism and, after obtaining consent, surveyed those youth and adults. Using a panel of households that was sampled based random direct dialing and physical addresses from the U.S. postal service, Ipsos built the KnowledgePanel. The KnowledgePanel consists of approximately 60,000 people who are representative of residents across the U.S. on the basis of geography, age, gender, race, education, and income but not necessarily Autism or other disability status. Surveys are administered online, and internet capable devices and internet service is provided by Ipsos if the family does already have access at recruitment. If an Autistic youth was considered a minor, a parent/caregiver provided consent, and the youth was asked for assent. If there was more than one Autistic youth in the household, the survey was programmed to randomly select a potential participant. This study is part of a larger study that also examined outcomes of employment and education and collected responses from both Autistic people and parents/caregivers of Autistic youth. All participants included in this study were expected to respond for themselves. All data were collected in the fall of 2023.

### 2.2 Survey

Researchers, with the support of a panel of subject matter experts that was inclusive of Autistic individuals, designed a survey to ask about both experiences in secondary school (predictors) and life after secondary school (outcomes). Research on predictors of postschool outcomes (11, 12, 23, 24) guided the development of questions about transition activities. The predictors can be grouped into the four clusters, as identified in the Predictor Implementation School/District Self-Assessment (PISA) (33): career development, policy, collaborative systems, and student skills. For the purposes of this study, the researchers focused on predictors on the collaborative systems and student skills clusters. The collaborative systems predictor was student support, which was conceptualized in this study as social support. Student skills predictors include community experiences, goal setting, psychological empowerment, self-advocacy/self-determination, self-care, self-realization, social skills, travel skills, technology skills, and youth-autonomy/decision-making. The commonly researched outcome of living independently was added as an outcome question along with other possible life experience outcomes of pregnancy (self or partner), being a caregiver, falling in love, marriage, and being arrested. Demographic (age, gender, race/ethnicity) and proxies for adaptive behavior (physical health problems, mental health problems, and receipt of vocational services, personal assistance, personal organization services, and case management) questions were added to both describe the participants and control for factors that might influence life experiences. See **Supplementary Table 1** for details on question wording and response choices.

### 2.3 Statistical analyses

After examining the data set (34) for missing data (see **Supplementary Material**), multivariate imputation by chained equations (MICE) (35) was implemented to handle missing data in R (36) (RRID: SCR_001905) to generate 40 imputed datasets (see **Supplementary Table 1** for amount missing from predictor variables). In most cases, only 2 (0.7%) or 3 (1.1%) responses were missing with the largest amount of missingness for the question about having a case manager (9.9%) (see **Supplementary Table 1**). Enders recommends a minimum of 20 imputed data sets, and more if the fraction missing values are high. With the largest fraction missing value of 25.5% for the community experiences question, the minimum recommended number of imputed data sets was doubled to 40. All collected variables were used to predict missing values with variables imputed from the least amount of missingness to the most amount of missingness, and iterations was set to 10. After imputation, the imputed data was examined for convergence (imputed lines are intermingled) to ensure that sufficient iterations were used in the process to produce independent results (37). Weights were applied to all analyses so that inferences could be made to the general population of Autistic people in the U.S. provided those individuals could also respond to such a survey without support.

To support interpretation of the results, behavior problems was reverse coded so that high ratings indicated fewer problems. The travel skills question was also recoded to indicate higher scores meant higher skills. Ratings for all other predictor questions were already coded to indicate more experience or higher skills as ratings increased. No proxies for adaptive behavior were recoded.

The analyses were all correlational in nature with no claim of causality. All outcomes were asked as ‘yes/no’ questions, so the binary responses were analyzed as logistic regression models with estimates reported as odds. To enable comparison against other research in the field (38, 39), each model contained demographics, proxies for adaptive behaviors, and one predictor. All models were estimated in Mplus (40) (RRID: SCR_015578) with all six outcomes estimated at once. The regression models took the following form: *log(odds) = β_0_ + β_1_ × Predictor*


$$LE_{i}=\beta_{0}+\beta_{1}\mathrm{Predictor}+\beta_{d}\mathrm{Demographics}+\beta_{ab}\mathrm{AB}+\varepsilon_{i}$$

*LE_i_* refers to the life experience outcomes and *β_0_* is the intercept term. *β_1_* is the estimate for each collaborative support or student skill predictor. *β_d_* refers to the collection of demographic variables tested in the model, and *β_ab_* for the adaptive behavior proxies. Lastly, *ϵ_i_* refers to the error term. The original log odds estimates were converted to odds by taking the exponent of each value. The only exception to this description was for the estimation of being arrested for participants who took the Spanish language version of the survey as they all responded ‘No’ to that question.

Values greater than 1.0 indicated increased odds of experiencing outcomes, and values less than 1.0 indicate reduced odds of an outcome. The more extreme the value is above 1.0, the stronger the relationship is between the predictor and odds that the outcome will occur. Values less than 1.0 indicate reduced odds of an outcome happening with values closer to 0 indicating very low odds of the event happening. Statistically significant odds will not contain 1.0 in the confidence interval, and confidence intervals with a large range should be interpreted with caution. The *a priori* alpha level was set to.05 to determine significance. P-values were included in the text with 95% confidence intervals for all estimates provided in the tables.

## 3 Results

### 3.1 Sample characteristics

Of the 60,000 KnowledgePanel members that were invited to take the screening survey, responses were received from 39,289 individuals, and 860 were considered eligible as an Autistic person or parent/caregiver of an Autistic youth. A total of 272 participants who could take the survey without support consented or assented to participate. Respondents ranged in age from 15 to 84 years (M = 29.2, SD = 14.5); 98 were minors and 174 were adults. As expected with this population, data was collected from more males (n = 152) than females (n = 120). The largest group of respondents identified as White (n = 157) and the smallest group identified as African American (n = 16). Eight participants chose to take the survey in Spanish. See **Table 1** for unweighted and weighted counts and percentages.

**Table 1:** Sample description with and without weights applied.

	n	%	Weighted n	Weighted %
Participants	272	100.0	272.0	100.0
Age Group				
Adult (18-84)	174	64.0	174.0	64.0
Youth (15-17)	98	36.0	98.0	36.0
Spanish-language version	8	2.9	8.4	3.1
Sex				
Male	152	55.9	166.2	61.1
Female	120	44.1	105.8	38.9
Race/Ethnicity				
African American	16	5.9	24.2	8.9
Hispanic/Latino	47	17.3	47.6	17.5
Multiple races	19	7.0	13.6	5.0
White	157	57.7	145.8	53.6
Other, non-Hispanic	33	12.1	40.8	15.0

### 3.2 Life experience rates

Of the six life experience questions that were asked, the highest rating was for falling in love (43%). Almost a quarter had moved into their own home and one in five was a caregiver for a child or another family member. The lowest rated experience was being arrested (10%) (see **Table 2** for rates and standard deviations). Because some of these life experiences may be less common for Autistic youth still in high school, rates were calculated for youth, adults, and then overall. For youth, the most common experience was having fallen in love (31%) followed by being a caregiver (8%). For adults, 50% reported having fallen in love and 41% reported getting married. The next most frequent experience was moving into one’s own home (32%).

**Table 2:** Rates of life experiences.

	Youth		Adults		Total	
	M	SD	M	SD	M	SD
Pregnancy (yourself or your partner)	0.05	0.22	0.21	0.41	0.16	0.36
Was a caregiver for child or other family member	0.08	0.28	0.23	0.42	0.19	0.39
Fallen in love	0.31	0.46	0.50	0.50	0.43	0.50
Got married	0.07	0.25	0.41	0.43	0.18	0.39
Moved into your own home	0.07	0.25	0.32	0.47	0.24	0.43
Arrested	0.05	0.21	0.13	0.34	0.10	0.30

### 3.3 Demographics and proxies for adaptive behavior

For demographics variables of the participants, only age and sex were related to our life experience variables (see **Tables 3**, **4**). Age was related to increased odds for pregnancy (OR = 1.12, p <.001), caregiving (OR = 1.08, p = .006), falling in love (OR = 1.07, p = .002), marriage (OR = 1.14, p <.001), living independently in one’s own home (OR = 1.16, p <.001), and being arrested (OR = 1.08, p = .005), values that are multiplicative as someone grows older. Age as a quadratic term, with the hypothesis that change over time is not linear, was tested but was not significant for all tested outcomes; OR values all equaled 1.00 after rounding to two decimal places with standard errors of 0.001 or 0.002 and 95% confidence intervals essentially ranging from 1.00 to 1.00. Identifying as female was related to marriage (OR = 2.49, p = .041) and pregnancy (OR = 2.43, p = .045) with odds for females more than twice as large than males. All respondents who took the Spanish-language version of the survey indicated they had never been arrested so this outcome could not be tested for this subgroup and this variable was not a significant predictor for any outcome in this study. There were no significant differences related to a person’s race/ethnicity value in comparison to respondents who identified as White. As shown in **Tables 3**, **4**, reporting mental health or physical health problems that interfered with school or work were related to some outcomes, as was using organization services and having a case manager. Mental health problems were associated with increased odds of being a caregiver (OR = 2.64, p = .042), falling in love (OR = 4.41, p = .001), living in one’s own home (OR = 4.04, p = .015), and being arrested (OR = 17.24, p <.001). Respondents who reported physical problems were related to higher odds of being a caregiver (OR = 3.25, p = .015), marriage (OR = 2.81, p = .040), and living outside of the family home (OR = 3.61, p = .009). Using organization services was related to increased odds of being arrested (OR = 3.74, p = .038). Having a case manager was related to reduced odds of marriage (OR = 0.31, p = .035), living outside of the family home (OR = 0.34, p = .043), and being arrested (OR = 0.28, p = .048).

**Table 3:** Odds Ratios (OR) and confidence interval (CI) of demographics and proxies for adaptive behavior for pregnancy, caregiving, and falling in love.

Variable	Pregnancy	95% CI	Care-giving	95% CI	Fallen in love	95% CI
Demographics						
Age, centered	1.12	[1.06, 1.18]	1.08	[1.02, 1.14]	1.07	[1.03, 1.12]
Female	2.43	[1.02, 5.80]	1.31	[0.60, 2.86]	1.37	[0.63, 2.96]
Spanish language version	2.59	[0.43, 15.59]	2.27	[0.37, 13.79]	5.67	[0.65, 49.35]
Race/ethnicity						
African American	1.12	[0.27, 4.63]	0.41	[0.08, 2.05]	0.66	[0.13, 3.33]
Other, non-Hispanic/Latino	1.50	[0.21, 10.64]	1.19	[0.25, 5.73]	1.40	[0.37, 5.38]
Hispanic/Latino	0.75	[0.25, 2.26]	0.71	[0.28, 1.79]	0.81	[0.32, 2.04]
Multi-racial	3.06	[0.28, 33.36]	1.57	[0.20, 12.50]	2.90	[0.74, 11.38]
Adaptive behavior proxies						
Mental health problems	3.29	[0.88, 12.27]	2.64	[1.04, 6.75]	4.41	[1.87, 10.40]
Physical health problems	2.24	[0.79, 6.36]	3.25	[1.26, 8.42]	1.18	[0.47, 2.96]
Vocational services	2.24	[0.81, 6.21]	1.86	[0.66, 5.22]	2.73	[0.88, 8.49]
Personal assistance	0.91	[0.26, 3.23]	0.57	[0.17, 1.98]	0.95	[0.22, 4.19]
Organization services	1.22	[0.41, 3.65]	1.05	[0.25, 4.36]	0.76	[0.24, 2.47]
Case management	0.55	[0.19, 1.56]	0.57	[0.14, 2.38]	0.51	[0.17, 1.51]

**Table 4:** Odds Ratios (OR) and confidence interval (CI) of demographics and proxies for adaptive behavior for marriage, living in own home, and arrest.

	Marriage OR	95% CI	Home OR	95% CI	Arrest OR	95% CI
Demographics						
Age, centered	1.14	[1.08, 1.20]	1.16	[1.10, 1.22]	1.08	[1.02, 1.15]
Female	2.31	[1.04, 5.17]	1.73	[0.78, 3.86]	0.47	[0.12, 1.52]
Spanish language version	0.85	[0.08, 8.86]	0.58	[0.06, 5.63]	-–*	-–
Race/ethnicity						
African American	0.56	[0.12, 2.54]	0.49	[0.09, 2.66]	1.27	[0.20, 8.21]
Other, non-Hispanic/Latino	1.83	[0.23, 14.64]	1.4	[0.14, 13.96]	0.75	[0.09, 6.85]
Hispanic/Latino	0.82	[0.29, 2.29]	0.84	[0.31, 2.28]	2.14	[0.60, 7.67]
Multi-racial	2.38	[0.19, 30.57]	3.56	[0.55, 23.12]	0.13	[0.02, 1.11]
Adaptive behavior proxies						
Mental health problems	2.53	[0.75, 8.50]	4.04	[1.30, 12.53]	17.24	[3.31, 89.78]
Physical health problems	2.81	[1.05, 7.52]	3.61	[1.39, 9.39]	1.71	[0.39, 7.48]
Vocational services	1.71	[0.60, 4.87]	1.80	[0.53, 6.09]	1.51	[0.43, 5.28]
Personal assistance	1.27	[0.36, 4.51]	0.46	[0.10, 2.01]	1.88	[0.46, 7.71]
Organization services	1.43	[0.45, 4.48]	0.73	[0.21, 2.49]	3.74	[1.08, 12.97]
Case management	0.31	[0.10, 0.92]	0.34	[0.12, 0.97]	0.28	[0.08, 0.99]

*All respondents who took the Spanish-language version of the survey answered ‘no’ so having been arrested could not be tested.

### 3.4 Social support and student skills

Social support, also referred to as student support for those in secondary settings, was only related to falling in love (OR = 1.58, 95% CI[1.03, 2.40], p = .034). The results were not significant for pregnancy (OR = 0.91, p = .747), caregiving (OR = 1.21, p = .43), marriage (OR = 1.24, p = .383), living outside of the family home (OR = 1.48, p = .113), or being arrested (OR = 0.78, p = .440).

Unlike social support, every life experience outcome was related to one or more student skills. The results described in this section are organized by outcome variable with student skills alphabetized in the table. In the narrative that follows, predictors that are related to the broad concept of self-determination are described together, if applicable. Note, community experiences, grooming, social skills (i.e., control temper when arguing, social skills, persistence, behavior problems) were only related to being arrested.

Setting goals to work outside the home was related to increased odds of pregnancy (OR = 3.03, p = .040) while ratings on technology skills were related to reduced odds (OR = 0.63, p = .015). No other student skills were significant predictors of pregnancy (see **Table 5**).

**Table 5:** Odds Ratios (OR) and 95% confidence intervals (CI) of student skills to predict pregnancy, caregiving, and falling in love.

	Pregnancy OR	95% CI	Care-giving OR	95% CI	Fallen in love OR	95% CI
Community experiences	0.91	[0.28, 2.92]	1.46	[0.57, 3.72]	1.66	[0.62, 4.42]
Goal setting						
Postsecondary education	1.53	[0.64, 3.64]	1.57	[0.68, 3.63]	1.85	[0.86, 4.00]
Work outside of the house	3.03	[1.05, 8.73]	2.83	[0.95, 8.37]	3.38	[1.50, 7.65]
Live outside of family home	2.40	[0.92, 6.25]	6.91	[2.50, 19.07]	3.93	[1.77, 8.74]
Make friends, find hobbies	1.40	[0.53, 3.68]	2.26	[0.86, 5.93]	1.97	[0.90, 4.32]
Psychological empowerment	1.35	[0.89, 2.04]	2.00	[1.20, 3.32]	1.85	[1.22, 2.81]
Self-advocacy/self-determination	1.35	[0.90, 2.03]	1.30	[0.93, 1.80]	2.09	[1.53, 2.85]
Self-care						
Household tasks	1.47	[0.98, 2.21]	1.60	[1.11, 2.30]	1.65	[1.21, 2.25]
Grooming	1.25	[0.82, 1.91]	1.31	[0.93, 1.86]	1.32	[0.90, 1.91]
Self-realization	1.11	[0.93, 1.32]	1.13	[0.98, 1.30]	1.15	[1.00, 1.32]
Social skills						
Join activities	1.15	[0.80, 1.66]	0.91	[0.67, 1.25]	1.36	[1.00, 1.85]
Behavior causes problems*	0.93	[0.64, 1.36]	0.73	[0.49, 1.08]	0.83	[0.59, 1.17]
Control temper when arguing	1.44	[0.94, 2.20]	1.15	[0.81, 1.63]	0.99	[0.73, 1.33]
Persist with tasks	1.11	[0.73, 1.68]	1.44	[1.03, 2.01]	1.66	[1.17, 2.37]
Technology skills	0.63	[0.43, 0.91]	0.83	[0.58, 1.19]	1.75	[1.21, 2.53]
Travel skills*	1.41	[0.92, 2.13]	1.15	[0.81, 1.63]	1.41	[1.05, 1.87]
Youth-autonomy/decision-making						
Interests	0.96	[0.67, 1.37]	0.96	[0.68, 1.35]	1.08	[0.79, 1.47]
Money	0.80	[0.56, 1.15]	1.49	[0.99, 2.24]	1.66	[1.15, 2.38]

*Predictor questions were reverse coded so odds > 1.0 would indicate fewer problems or higher skills.

Setting goals to live outside of the family home (OR = 6.91, p <.001), psychological empowerment (OR = 2.00, p = .008), self-care skills of being able to perform household tasks (OR = 1.60, p = .011), and social skills of persisting with tasks (OR = 1.44, p = .030) were all related in increased odds of becoming a caregiver (see **Table 5**).

The most significant findings were related to having fallen in love. Goal setting to both work outside the home (OR = 3.38, p = .003) and live outside the family home (OR = 3.93, p.001), psychological empowerment (OR = 1.85, p = .004), self-advocacy/self-determination (OR = 2.09, p <.001), making decisions about money (OR = 1.66, p = .006), being able to perform household tasks (OR = 1.65, p = .001), persistence with tasks (OR = 1.66, p = .005), and technology skills (OR = 1.75, p = .003) were all associated with increased odds of falling in love. Travel skills (OR = 1.41, p = .023) was also related to increased odds of having fallen in love. All results are shown in **Table 5**.

To a lesser degree, student skills were also related to marriage (see **Table 6**). Goal setting once again was related to the outcome for setting goals to live outside the family home (OR = 3.99, p = .007), along with psychological empowerment (OR = 1.84, p = .004), and social skills of persistence with tasks (OR = 1.59, p = .029). The social skills of controlling one’s temper (OR = 1.66, p = .012) when arguing was also significant with increased odds of being married.

**Table 6:** Odds Ratios (OR) and 95% confidence intervals (CI) of student skills to predict marriage, living in own home, and arrest.

	Marriage OR	95% CI	Home OR	95% CI	Arrest OR	95% CI
Community experiences	0.92	[0.29, 2.90]	1.77	[0.59, 5.26]	2.76	[0.95, 7.99]
Goal setting						
Postsecondary education	2.03	[0.85, 4.86]	3.10	[1.24, 7.74]	0.98	[0.24, 4.00]
Work outside of the house	2.25	[0.86, 5.88]	3.00	[1.19, 7.55]	1.04	[0.21, 5.24]
Live outside of family home	3.99	[1.46, 10.94]	6.07	[2.28, 16.17]	1.37	[0.41, 4.63]
Make friends, find hobbies	1.11	[0.41, 2.99]	1.29	[0.49, 3.42]	1.56	[0.40, 6.15]
Psychological empowerment	1.84	[1.22, 2.79]	1.68	[1.19, 2.38]	0.69	[0.41, 1.17]
Self-advocacy/self-determination	1.52	[0.99, 2.33]	1.31	[0.97, 1.77]	1.20	[0.83, 1.72]
Self-care						
Household tasks	1.37	[0.94, 1.98]	1.56	[1.09, 2.22]	1.11	[0.68, 1.83]
Grooming	1.03	[0.67, 1.56]	1.20	[0.78, 1.83]	0.82	[0.41, 1.62]
Self-realization	1.17	[0.98, 1.41]	1.13	[0.98, 1.32]	0.79	[0.63, 0.99]
Social skills						
Join activities	1.24	[0.90, 1.71]	1.25	[0.91, 1.71]	1.09	[0.67, 1.77]
Behavior causes problems*	0.87	[0.60, 1.26]	0.89	[0.60, 1.32]	0.61	[0.38, 0.98]
Control temper when arguing	1.66	[1.12, 2.46]	1.39	[0.95, 2.02]	0.56	[0.33, 0.95]
Persist with tasks	1.59	[1.05, 2.4]	1.33	[0.98, 1.82]	0.56	[0.35, 0.92]
Technology skills	0.97	[0.64, 1.46]	1.25	[0.80, 1.95]	1.19	[0.69, 2.05]
Travel skills*	1.27	[0.85, 1.9]	1.59	[1.07, 2.33]	1.38	[0.85, 2.24]
Youth-autonomy/decision-making						
Interests	1.33	[0.93, 1.91]	1.55	[1.06, 2.27]	0.88	[0.52, 1.48]
Money	0.96	[0.70, 1.30]	1.46	[1.08, 1.98]	0.70	[0.38, 1.29]

*Predictor questions were reverse coded so odds > 1.0 would indicate fewer problems or higher skills.

Living independently, or in one’s own home as defined in our survey, was related to eight different predictors (see **Table 6**). Setting goals for postsecondary education (OR = 3.10, p = .015), working outside the home (OR = 3.00, p = .020), and to live outside of one’s family home (OR = 6.07, p <.001) were all related to increased odds of living independently. That was also true for psychological empowerment (OR = 1.68, p = .003), self-care skills of doing household tasks (OR = 1.56, p = .014), as was making decisions about interests (OR = 1.55, p = .024) and spending money (OR = 1.46, p = .013). Travel skills (OR = 1.59, p = .020) were related to increased odds of living independently.

Being arrested was related to self-realization and several social skills (see **Table 6**). As self-realization increases, the odds of being arrested decrease (OR = 0.79, p = .043). Likewise, as behavior problems decrease, the odds of being arrested decrease (OR = 0.61, p = .040). Similarly, as ratings for controlling one’s temper when arguing (OR = 0.56, p = .031) and persisting with tasks go up, the related odds for being arrested decrease (OR = 0.56, p = .022).

## 4 Discussion

In this study we examined the rates of six types of life experiences, the degree to which personal characteristics and proxies for adaptive behavior related to those life experiences, and the effects of social support and student skills were associated with life experiences for Autistic individuals. We were able to confirm some findings in previous studies but found significant results in the domain of predictors of postschool outcomes (12), particularly student skills, thus adding to the literature.

### 4.1 Life experience rates

The highest rated life experience type was having fallen in love. Approximately one in five respondents reported they were married with this increasing to two in five for adults age 18 and older. The overall rate is similar to that identified by Mazurek (19), who studied a group of Autistic adults with a similar age range, but the rate was almost double for adult participants in this study. One in four participants (one in three adults) in this study reported that they were living in their own home. These rates are higher than those reported by Autistic youth in the NLTS2 (10) as well as for those receiving developmental disability services (13) and Autistic adults also diagnosed with an intellectual disability (14). But the values are still approximately half of the UK sample (16) when compared to the adults in this study. To date, there are no other known sources of information about Autistic people’s pregnancy rates for themselves or a partner or for becoming a caregiver for a child or other family member. These experiences were one in six and one in five respectively for Autistic people in this study, and slightly higher for just the adults. The overall reported rate for arrested was twice what was observed in the NLTS2 sample of Autistic youth, but this study included participants as old as 84 and the NLTS2 sample’s oldest participant was 22 (27).

### 4.2 Demographics and proxies for adaptive behavior

Some of this study’s findings on personal characteristics and proxies for adaptive behavior were confirmed. Like with the National Core Indicator participants (13), age was related to increased odds of living independently. We also found age as a significant predictor for having fallen in love, pregnancy, and marriage. Females also resulted in statistical differences with increased odds for pregnancy (upon being asked if the respondents have been pregnant or have gotten a partner pregnant) (41). There were also increased odds of getting married for females in comparison to males. Note, the lack findings for those who took the Spanish language version of the survey could be unique to this study due to the small number of participants. Aside from age as predictor, all of these other findings add to the literature for life experiences of Autistic people.

In this study, having a case manager was related to reduced odds of marriage and living in one’s own home. It is important to note that case management may be acting as a proxy for more complex support needs and therefore should not necessarily be interpreted as a causal effect of the service on these outcomes. No other known studies examined case management as a predictor, although in one study, the whole sample had case managers (13), so that could not have been tested. We also tested self-report of physical or mental problems and how they related to life experiences. Problems in these areas were related to increased odds of both caregiving and living in their own home. Although other studies provided some information about physical or mental health problems, few tested or reported findings related to these life experiences. Living situation was not related to overall health in a sample of NLST2 Autistic youth (10).

Other studies have identified differences in life experiences based on race and/or ethnicity (10, 20), but no statistical differences were identified in this study. Nor did we find any relationship between using vocational services or personal assistance services and any of the life experiences outcomes.

### 4.3 Social support and student skills

The primary focus of this study was to identify which predictors of postschool outcomes related to life experiences, in particular social support and student skills. Our study confirmed the Shogren et al. (8) finding of the relationship between both psychological empowerment and autonomy, in the form of making decisions about interests, and making decisions about money, and independent living. Self-advocacy, a related skill not tested by Shogren et al. (8), was shown to be related to having fallen in love. Aside from these predictors, we also identified goal setting, household tasks, and travel skills as skills related to living in one’s own home.

Another way to examine the impact of transition experiences and skills on life experiences is to consider which variables were identified as statistically significant for multiple outcomes. Goal setting was broadly the most impactful skill that was tested. Goal setting was related to pregnancy, caregiving, having fallen in love, and marriage. Psychological empowerment was related to all of the outcomes except pregnancy and being arrested, and youth autonomy was related to having fallen in love. Social skills were related to the tested life experiences but to a lesser extent. Persistence with tasks was related to caregiving, having fallen in love, marriage, and being arrested while controlling one’s temper when arguing was related to marriage and being arrested. Fewer behavior problems were related to reduced odds of being arrested, a similar finding to the NLTS2 sample (27).

In addition to predicting living in one’s own home, the skill of household tasks was related to caregiving and having fallen in love. Travel skills was also related to having fallen in love. Technology skills were related to increased odds of having fallen in love but the odds were reduced for having been pregnant or getting a partner pregnant. The odds were also increased for having fallen in love for those who were able to get places on their own during high school. Aside from the research that confirmed some of the self-determination findings (8), all other findings add to the literature on predictors of adult life experiences.

### 4.4 Implications for practice

Many of the same student skills that are predictive of postschool outcomes in education and employment (12) also apply to life experiences, as shown in this study. Thus, practitioners have an opportunity to both design and deliver transition programs that will expand the possibilities for full life participation, in particular adding or strengthening activities that are related to more than one type of life experience, like goal setting. Hopefully, this broader perspective will then in turn have a positive impact on the quality of life for Autistic youth and adults as they imagine a bigger future for themselves. In this study, we linked several skills related to self-determination (e.g., goal setting, psychological empowerment, youth autonomy/decision-making) with outcomes related to life experiences. Given this understanding, practitioners are encouraged to teach their Autistic students specific skills related to self-determination using effective practices, such as the Self-Determined Learning Model of Instruction (SDLMI) (41). The SDLMI, as an example, is an evidence-based practice that promotes goal setting, decision making, and other self-determined skills through a student-directed process of setting, working toward, and monitoring progress on goals. Given the flexibility of the SDLMI, practitioners could identify goal categories for students related to life experiences, and students could then set their own goals related to this outcome area. Self-determination skill building can also be incorporated into sexual health curricula, to encourage young Autistic people’s autonomy and abilities to speak up for their wants and needs in socially appropriate ways when building and maintaining social and sexual relationships, given that only half of our respondents aged 15-84 (with an average age of 29) had never fallen in love and less than half had gotten married, experienced pregnancy (self or partner), or served as a caregiver. It is imperative that sexual health curricula for Autistic youth be equitable, accessible, and culturally responsive (42). Lastly, teaching social skills are important for positive outcomes but also for possibly reducing odds of being arrested. The literature also recognizes that police also need to be provided information and trained. A scoping review of this research found that trainings focused on information, recognizing behaviors, and engagement strategies. Some studies included an Autistic person in the design of the program, but not all. Including Autistic people in the design and delivery of police training is recommended (43).

Practitioners should have an understanding of their students’ cultures (44), as some types of life experiences, like living on one’s own, may not be as valued or desirable by individual students and/or their family’s cultures. Cultural and linguistic diversity may also shape participation in different experiences, such as for students whose native language is different from the majority language in their country. Practitioners should acknowledge and respect the youth’s preferences and that of their families’ when supporting the youth through their postsecondary transition. Some Autistic individuals may not desire certain milestones (e.g., marriage, living independently), and their choices to not pursue certain milestones should not be framed as deficits. It is encouraged that practitioners support Autistic youth in determining which life experiences are important to them and for them and to help these youth build skills that will help them achieve their desired outcomes.

### 4.5 Limitations and future research

The survey was administered to a nationally representative sample based on geography, income, age, gender and race/ethnicity, but it is unclear as to the representativeness based on the degree of support needed to answer the survey. For this study, participants were asked to respond for themselves, an activity that may not be possible for those with the highest level of support needs, limiting the generalizability of the results to the whole population of Autistic people in the U.S (45). Also, some respondents were asked to answer questions about high school experiences that were decades in the past. Based on research that compared self-report to administrative data, after a year self-reports become unreliable (46). This unreliability may result in significant findings when not present (Type I) or not identifying significant predictors when present in the model (Type II errors) (47). With that uncertainty in the data, there is potential bias in the results so this research should be replicated with other samples and additional sources of information considered, including administrative data when possible. Lastly, one another outcome was tested (adjudicated or convicted), and we could not evaluate it due to the small incidence rate in our sample.

With so little research in the area of life experiences, there are many directions to take this research. Aside from replication of this study and particularly larger samples from different cultural groups, as some estimates obtained in this study may be due to some small subgroups. Meaningful types of life experiences should also be defined and tested against predictors of postschool outcomes. We understand that a person can engage in a wide range of life experiences after high school and that the list we tested was limited. Future research should explore other life experiences (e.g., getting a driver’s license, registering to vote) not investigated in the current study. Our study also focused primarily on positive life experiences. Future research should consider exploring factors that predict negative outcomes (e.g., getting divorced or going to jail) after high school as well. Given the unreliability of self-report, researchers should also explore ways to triangulate the experiences and outcomes of Autistic students and youth so more reliable data can be collected and tested.

### 4.6 Conclusion

There are a range of life experiences a person can have in adulthood. To date, however, much of the literature on life experiences for Autistic people is more than a decade old and largely focused on living independently, an outcome that may not be desired or valued by certain individuals. Through this study, we identified rates of life experiences, how life experiences are related to personal characteristics, and the degree to which skills Autistic youth build in school relate to life experiences. The adult research on the impact of life experiences on an Autistic person’s quality of life is limited, although independent living is often conceptualized as having a high quality of life in the transition predictor literature. Future research should explore a broad range of life experiences for Autistic people, with input on these life experiences provided from Autistic people, their family members, and others with expertise in Autistic people’s transition to adulthood. That research should also include Autistic people with all levels of support needs.

## Data Availability

The original contributions presented in the study are publicly available. This data can be found here: https://osf.io/ueay6/overview?view_only=71fa3968cd1949b8962fb34df1affc0c.

## References

[1] Ayres M Parr JR Rodgers J Mason D Avery L Flynn D . A systematic review of quality of life of adults on the autism spectrum. Autism. (2018), 774–83. doi: 10.1177/1362361317714988, PMID: 28805071

[2] Burgess AF Gutstein SE . Quality of life for people with autism: Raising the standard for evaluating successful outcomes. Child Adolesc Ment Health. (2007), 80–6. doi: 10.1111/j.1475-3588.2006.00432.x, PMID: 32811109

[3] Chiang HM Wineman I . Factors associated with quality of life in individuals with autism spectrum disorders: A review of literature. Res Autism Spectr Disord. (2014), 974–86. doi: 10.1016/j.rasd.2014.05.003, PMID: 41935531

[4] Ikeda E Hinckson E Krägeloh C . Assessment of quality of life in children and youth with autism spectrum disorder: a critical review. Qual Life Research: Int J Qual Life Aspects Treatment Care Rehabilitation. (2014), 1069–85. doi: 10.1007/s11136-013-0591-6, PMID: 24310317

[5] Whiteneck G Dijkers MP . Difficult to measure constructs: conceptual and methodological issues concerning participation and environmental factors. Arch Phys Med Rehabil. (2009) 90:S22–35. doi: 10.1016/j.apmr.2009.06.009, PMID: 19892071

[6] World Health Organization . International classification of functioning, disability and health. Geneva: World Health Organization (2001). Available online at: https://www.who.int/classifications/international-classification-of-functioning-disability-and-health (Accessed March 5, 2026).

[7] Cameron LA Borland RL Tonge BJ Gray KM . Community participation in adults with autism: A systematic review. J Appl Res Intellectual Disabilities. (2022), 421–47. doi: 10.1111/jar.12970, PMID: 34907624

[8] Shogren KA Lee J Panko P . An examination of the relationship between postschool outcomes and autonomy, psychological empowerment, and self-realization. J Special Education. (2017) 51:115–24. doi: 10.1177/0022466916683171, PMID: 41930703

[9] Nasamran A Witmer SE Los JE . Exploring predictors of postsecondary outcomes for students with autism spectrum disorder. Source: Educ Training Autism Dev Disabilities. (2017) 52:343–56. doi: 10.1177/215416471705200402, PMID: 41930703

[10] Anderson KA Shattuck PT Cooper BP Roux AM Wagner M . Prevalence and correlates of postsecondary residential status among young adults with an autism spectrum disorder. Autism. (2014) 18:562–70. doi: 10.1177/1362361313481860, PMID: 23996904 PMC4006316

[11] Test DW Mazzotti VL Mustian AL Fowler CH Kortering L Kohler P . Evidence-based secondary transition predictors for improving postschool outcomes for students with disabilities. Career Dev Exceptional Individuals. (2009) 32:160–81. doi: 10.1177/0885728809346960, PMID: 41930703

[12] Mazzotti VL Rowe DA Kwiatek S Voggt A Chang WH Fowler CH . Secondary transition predictors of postschool success: an update to the research base. Career Dev Transit Except Individ. (2021) 44:47–64. doi: 10.1177/2165143420959793, PMID: 41930703

[13] Hewitt AS Stancliffe RJ Hall-Lande J Nord D Pettingell SL Hamre K . Characteristics of adults with autism spectrum disorder who use residential services and supports through adult developmental disability services in the United States. Res Autism Spectr Disord. (2017) 34:1–9. doi: 10.1016/j.rasd.2016.11.007, PMID: 41935531

[14] Farley M Cottle KJ Bilder D Viskochil J Coon H McMahon W . Mid-life social outcomes for a population-based sample of adults with ASD. Autism Res. (2018) 11:142–52. doi: 10.1002/aur.1897, PMID: 29266823 PMC5924705

[15] Gray KM Keating CM Taffe JR Brereton AV Einfeld SL Reardon TC . Adult outcomes in autism: community inclusion and living skills. J Autism Dev Disord. (2014) 44:3006–15. doi: 10.1007/s10803-014-2159-x, PMID: 24915930

[16] Mason D McConachie H Garland D Petrou A Rodgers J Parr JR . Predictors of quality of life for autistic adults. Autism Res. (2018) 11:1138–47. doi: 10.1002/aur.1965, PMID: 29734506 PMC6220831

[17] Povenmire-Kirk TC Lindstrom L Bullis M . De escuela a la vida adulta/from school to adult life: Transition needs for Latino youth with disabilities and their families. Career Dev Exceptional Individuals. (2010) 33:41–51. doi: 10.1177/0885728809359004, PMID: 41930703

[18] Lin LY Huang PC . Quality of life and its related factors for adults with autism spectrum disorder. Disabil Rehabil. (2019) 41:896–903. doi: 10.1080/09638288.2017.1414887, PMID: 29228834

[19] Mazurek MO . Loneliness, friendship, and well-being in adults with autism spectrum disorders. Autism. (2014) 18:223–32. doi: 10.1177/1362361312474121, PMID: 24092838

[20] Myers E Davis BE Stobbe G Bjornson K . Community and social participation among individuals with autism spectrum disorder transitioning to adulthood. J Autism Dev Disord. (2015) 45:2373–81. doi: 10.1007/s10803-015-2403-z, PMID: 25725812

[21] Will M . OSERS Programming for the transition of youth with disabilities: Bridges from school to working life (1983). Available online at: http://files.eric.ed.gov/fulltext/ED256132.pdf (Accessed January 22, 2026).

[22] Rusch FR Allenphelps L . Secondary special education and transition from school to work: A national priority. Except Child. (1987) 53:487–92. doi: 10.1177/001440298705300601, PMID: 2953604

[23] Haber MG Mazzotti VL Mustian AL Rowe DA Bartholomew AL Test DW . What works, when, for whom, and with whom: A meta-analytic review of predictors of postsecondary success for students with disabilities. Rev Educ Res. (2016) 86:123–62. doi: 10.3102/0034654315583135, PMID: 38293548

[24] Mazzotti VL Rowe DA Sinclair J Poppen M Woods WE Shearer ML . Predictors of post-school success: A systematic review of NLTS2 secondary analyses. Career Dev Transition Exceptional Individuals. (2016) p:196–215. doi: 10.1177/2165143415588047, PMID: 41930703

[25] Rowe DA Alverson CY Unruh DK Fowler CH Kellems R Test DW . A Delphi study to operationalize evidence-based predictors in secondary transition. Career Dev Transit Except Individ. (2015) 38:113–26. doi: 10.1177/2165143414526429, PMID: 41930703

[26] Test DW Coyle J Rusher D Carter E Seaman-Tullis R Odom SL . Secondary transition of students with autism spectrum disorder. Educ Train Autism Dev Disabil. (2020) 55:247–63. doi: 10.1177/215416472005500302, PMID: 41930703

[27] Rava J Shattuck P Rast J Roux A . The prevalence and correlates of involvement in the criminal justice system among youth on the autism spectrum. J Autism Dev Disord. (2017) 47:340–6. doi: 10.1007/s10803-016-2958-3, PMID: 27844248

[28] Tint A Palucka AM Bradley E Weiss JA Lunsky Y . Correlates of police involvement among adolescents and adults with autism spectrum disorder. J Autism Dev Disord. (2017) 47:2639–47. doi: 10.1007/s10803-017-3182-5, PMID: 28612245

[29] American Psychiatric Association . Diagnostic and statistical manual of mental disorders: DSM-5. 5th ed. Washington, DC: American Psychiatric Association (2013). doi: 10.1176/appi.books.9780890425787, PMID:

[30] U.S. Centers for Disease Control and Prevention . Data and statistics on autism spectrum disorder. Atlanta: U.S. Centers for Disease Control and Prevention (2025). Available online at: https://www.cdc.gov/autism/data-research/index.html (Accessed March 8, 2026).

[31] Individuals with Disabilities Education Improvement Act of 2004. Pub. L. No. 108-446, United States of America (2004): 108–446.

[32] American Psychiatric Association . Diagnostic and statistical manual of mental disorders. fifth edition. Washington, DC: American Psychiatric Association Publishing (2022). p. 1050.

[33] National Technical Assistance Center on Transition: The Collaborative . Predictor implementation school/district self-assessment. (2023). Available online at: https://transitionta.org/pisa-self-assessment/ (Accessed March 5, 2026).

[34] Shaw LA Brendli Brown K Enayati H . National survey. (2023).

[35] Buuren SV Groothuis-Oudshoorn K . mice: multivariate imputation by chained equations in R. J Stat Softw. (2011) 45:1–67. doi: 10.18637/jss.v045.i03

[36] R Core Team . R: A language and environment for statistical computing. Vienna: Foundation for Statistical Computing (2024).

[37] Enders CK . Applied missing data analysis. New York: Guilford Press (2010). p. 377.

[38] Chiang HM Cheung YK Li H Tsai LY . Factors associated with participation in employment for high school leavers with autism. J Autism Dev Disord. (2013) 43:1832–42. doi: 10.1007/s10803-012-1734-2, PMID: 23224594

[39] Chiang HM Cheung YK Hickson L Xiang R Tsai LY . Predictive factors of participation in postsecondary education for high school leavers with autism. J Autism Dev Disord. (2012) 42:685–96. doi: 10.1007/s10803-011-1297-7, PMID: 21618065

[40] Muthén LK Muthén BO . Mplus: statistical analysis with latent variables. Los Angeles: Muthén & Muthén (2017).

[41] National Center for Health Statistics CDC/National Center for Health Statistics/Division of Analysis and Epidemiology . Births [Figure 2. Birth rates, by race and Hispanic origin of mother. United States (2023). Available online at: https://www.cdc.gov/nchs/hus/topics/births.htm (Accessed December 14, 2025).

[42] Shogren KA Raley SK Burke KM Wehmeyer ML . The self-determined learning model of instruction teacher’s guide. Lawrence: Kansas University Center on Developmental Disabilities (2019).

[43] Dickerson A Bond KT . Enhancing sexual health education for autistic youth: A scoping review of barriers, gaps, and solutions. Am J Sex Educ. (2025). doi: 10.1080/15546128.2025.2552489, PMID: 41179930 PMC12574449

[44] Sreckovic MA Kenney CK Wallace M . Autism training for law enforcement officers: A scoping review. J Autism Dev Disord. (2023) 53:3835–46. doi: 10.1007/s10803-022-05692-y, PMID: 35925431

[45] Short ME Goetzel RZ Pei X Tabrizi MJ Ozminkowski RJ Gibson TB . How accurate are self-reports? Analysis of self-reported health care utilization and absence when compared with administrative data. J Occup Environ Med. (2009) 51:786–96. doi: 10.1097/JOM.0b013e3181a86671, PMID: 19528832 PMC2745402

[46] Trainor AA Carter EW Karpur A Martin JE Mazzotti VL Morningstar ME . A framework for research in transition: identifying important areas and intersections for future study. Career Dev Transit Except Individ. (2020) 43:5–17. doi: 10.1177/2165143419864551, PMID: 41930703

[47] Cohen J Cohen P West SG Aiken LS . Applied multiple regression/correlation analysis for the behavioral sciences. 3rd ed. New York: Routledge (2015). p. 703.

