# Supplementary material for: Autistic people and life experiences: the role of student skills and support
Source: Front Psychiatry. 2026 Apr 10;17:1733550. doi: 10.3389/fpsyt.2026.1733550 (PMC13106597; doi:10.3389/fpsyt.2026.1733550)
Supplement: Supplementary file 1 [file Table1.docx]

**Supplementary Table**

Table S1. Survey items with percent (%) missing

| Variable Type | Short name | Survey item | Response Choices | % Missing |
| --- | --- | --- | --- | --- |
| Outcome | Pregnancy | Did you have any of these other experiences? Pregnancy (yourself or your partner) | binary (0-1) | 0.7 |
| Outcome | Caregiver | Did you have any of these other experiences? Was a caregiver for child or other family member | binary (0-1) | 1.1 |
| Outcome | Fallen in love | Did you have any of these other experiences? Fallen in love | binary (0-1) | 0.0 |
| Outcome | Marriage | Did you have any of these other experiences? Got married | binary (0-1) | 0.7 |
| Outcome | Home | Did you have any of these other experiences? Moved into your own home | binary (0-1) | 0.0 |
| Outcome | Arrested | Did you have any of these other experiences? Arrested | binary (0-1) | 0.7 |
| Predictor | Social support | While in high school, how many people (like family, friends, and teachers) helped you prepare for life after high school? | 5-point Likert-type scale (0-4) | 0.7 |
| Predictor | Community experiences | Did you apply job-specific skills you learned in high school to places outside of school? | binary (0-1) | 5.9 |
| Predictor | Goal setting - Postsecondary education | While in high school, did you set goals to - Attend a 2- or 4-year college or a vocational training program | binary (0-1) | 1.1 |
| Predictor | Goal setting - Work outside of the house | While in high school, did you set goals to - Work outside of the house | binary (0-1) | 0.7 |
| Predictor | Goal setting - Live outside of family home | While in high school, did you set goals to - Live outside of my parent or guardian’s house | binary (0-1) | 0.7 |
| Predictor | Goal setting - Make friends, find hobbies | While in high school, did you set goals to - Make friends or find hobbies after high school | binary (0-1) | 1.1 |
| Predictor | Psychological empowerment | Make your own decisions | 5-point Likert-type scale (0-4) | 1.1 |
| Predictor | Self-advocacy/self-determination | How comfortable are you speaking up to get the services you needed to prepare for getting or keeping a job? | 5-point Likert-type scale (0-4) | 0.7 |
| Predictor | Self-care - household tasks | How well are you able to independently complete everyday household tasks such as laundry, fixing a lunch, and cleaning? | 5-point Likert-type scale (0-4) | 0.0 |
| Predictor | Self-care - grooming | How well are you able to independently maintain your own personal grooming and hygiene? | 5-point Likert-type scale (0-4) | 0.4 |
| Predictor | Self-realization |  | Sum score of self-realization items | 1.1 |
| Predictor | Self-realization | How often would you agree with the following statements? I knew what I was good at | 5-point Likert-type scale (0-4) | 0.4 |
| Predictor | Self-realization | How often would you agree with the following statements? I liked myself | 5-point Likert-type scale (0-4) | 0.7 |
| Predictor | Self-realization | How often would you agree with the following statements? I was confident in my abilities | 5-point Likert-type scale (0-4) | 1.1 |
| Predictor | Social skills- Join activities | Thinking back to when you were in high school, how often did you - Join group activities without being told | 5-point Likert-type scale (0-4) | 0.7 |
| Predictor | Social skills- Behavior causes problems* | Thinking back to when you were in high school, how often did you - Behave at home in a way that caused problems for the family | 5-point Likert-type scale (0-4) | 1.1 |
| Predictor | Social skills- Control temper when arguing | Thinking back to when you were in high school, how often did you - Control your temper when arguing with peers | 5-point Likert-type scale (0-4) | 0.7 |
| Predictor | Social skills- Persist with tasks | Thinking back to when you were in high school, how often did you - Keep working at something until it is finished, even if it took a long time | 5-point Likert-type scale (0-4) | 0.7 |
| Predictor | Technology skills | While in high school, how good were you at using a computer? | 5-point Likert-type scale (0-4) | 0.7 |
| Predictor | Travel skills | While in high school, how well did you get places outside the home (like to school, to a nearby store or park, or to a neighbor’s house) on your own, without help? | 5-point Likert-type scale (1-5) | 4.4 |
| Predictor | Youth-autonomy/decision-making - interests | I participated in activities based on my career interests | 5-point Likert-type scale (0-4) | 0.7 |
| Predictor | Youth-autonomy/decision-making - money | I chose how to spend my money | 5-point Likert-type scale (0-4) | 1.1 |
| Control | Mental health | Did you have any of these health experiences? Mental health problem interfering with attending school or work | binary (0-1) | 0.4 |
| Control | Physical health | Did you have any of these health experiences? Physical health problems that interfered with attending school or work | binary (0-1) | 0.4 |
| Control | Vocational services | During the past 12 months, have you received any of the following services? Vocational services (like career counseling, help finding a job, training in job skills, job coaching, or vocational education) | binary (0-1) | 0.7 |
| Control | Personal assistance | During the past 12 months, have you received any of the following services? Personal assistance (like reader, interpreter, or respite care) | binary (0-1) | 0.4 |
| Control | Organizational services | During the past 12 months, have you received any of the following services? Planning or organizational services (to assist in planning your day or week, to assist in sequencing tasks) | binary (0-1) | 0.7 |
| Control | Case management | Do you have a case manager or someone who coordinates the services you receive? | binary (0-1) | 9.9 |
